# Mechano‐Chemiluminescent Hydrogel for Sustained Stress Visualization Under Mechanical Equilibrium

**DOI:** 10.1002/marc.202500256

**Published:** 2025-05-21

**Authors:** Yiwa Wang, Kou Okuro

**Affiliations:** ^1^ Department of Chemistry The University of Hong Kong Pokfulam Road Hong Kong 999077 China; ^2^ State Key Laboratory of Synthetic Chemistry The University of Hong Kong Pokfulam Road Hong Kong SAR 999077 China

**Keywords:** glucose oxidase, hydrogel, luminol reaction, mechano‐chemiluminescence, multivalent interactions

## Abstract

Mechanoluminescent materials serve as stress‐sensing platforms that emit light autonomously, featuring properties distinct from other mechanochromic materials for stress visualization. While these materials respond instantaneously to dynamic mechanical forces, visualization of stress distribution under static mechanical loads, where both mechanical and energetic equilibrium are established, remains a significant challenge in materials science, representing a critical issue in real‐time continuous stress monitoring. Here, an unprecedented mechano‐chemiluminescent hydrogel (^GOx^Gu‐gel) is presented that introduces a conceptually new approach to stress visualization. ^GOx^Gu‐gel contains glucose oxidase (GOx), which is inhibited by guanidinium (Gu^+^) ions in the network polymer through multivalent salt‐bridge interactions. Mechanical stress on ^GOx^Gu‐gel disrupts these multivalent interactions, liberating GOx to catalyze glucose oxidation, which generates hydrogen peroxide (H_2_O_2_) that then drives the luminol chemiluminescence. This novel mechanism successfully visualizes stress distribution under sustained static loads, addressing the key limitation of conventional mechanoluminescent materials. The luminescence can be repeatedly switched on/off through multiple stress cycles, with original performance recovered through simple substrate replenishment.

## Graphical abstract

A mechano‐chemiluminescent hydrogel (^GOx^Gu‐gel), unprecedentedly visualizes internal stress under static mechanical loads. Unlike conventional mechanoluminescent materials that detect only dynamic forces, ^GOx^Gu‐gel continuously emits light under static forces via the reversible liberation of glucose oxidase (GOx) from multivalent guanidinium ion (Gu^+^) inhibition, enabling GOx‐mediated glucose oxidation that drives luminol chemiluminescence.

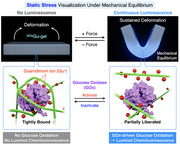

## 1 Introduction

**Figure 1:**
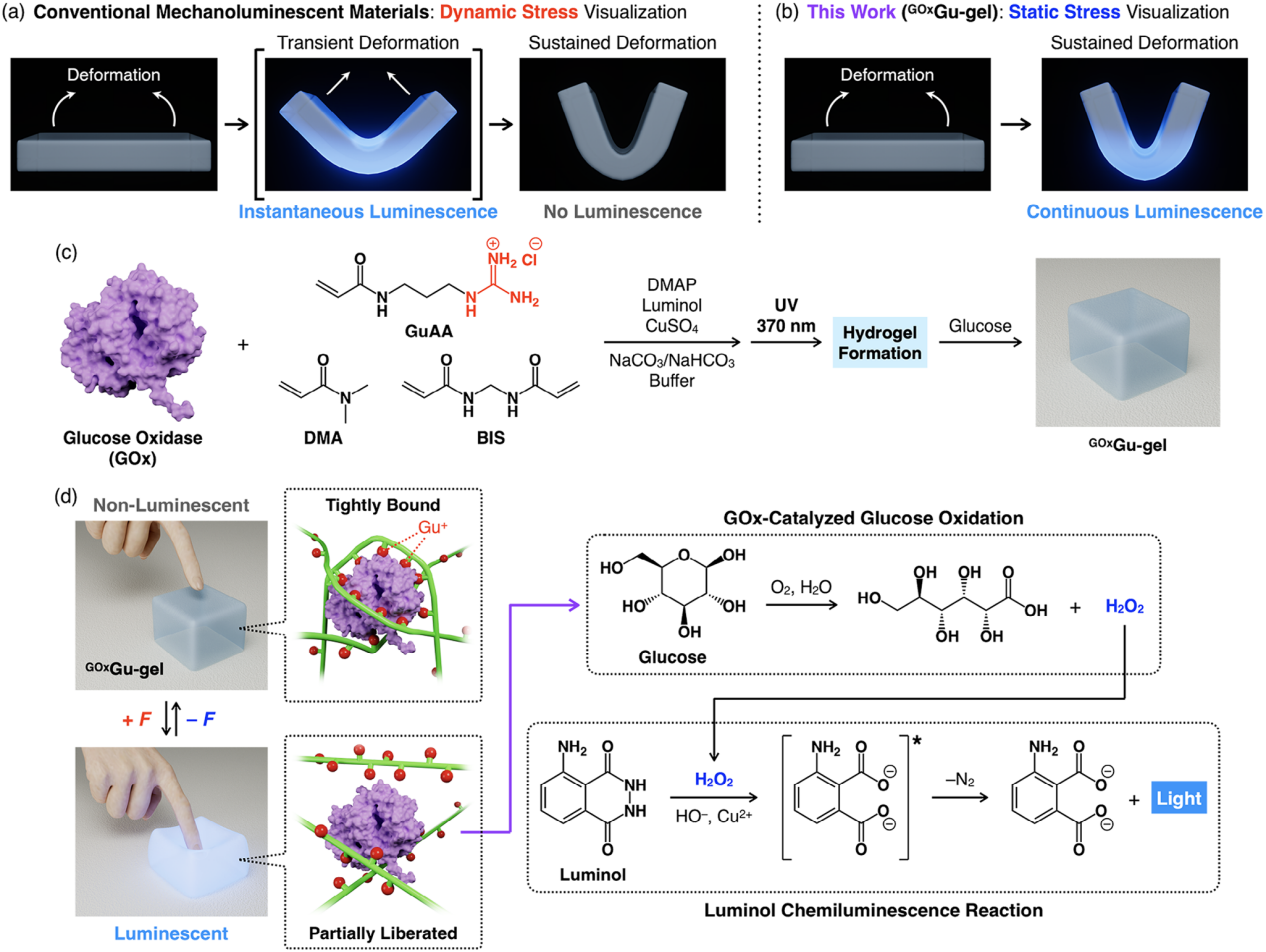
a,b) Schematic illustrations of a) dynamic stress visualization by conventional mechanoluminescent materials and b) static stress visualization by the mechano‐chemiluminescent hydrogel (^GOx^Gu‐gel) developed in this study. c) Preparation of ^GOx^Gu‐gel: *N*,*N*‐dimethylacrylamide (DMA), a guanidinium ion (Gu^+^)‐functionalized acrylamide derivative (GuAA), and *N*,*N'*‐methylenebis(acrylamide) (BIS) are combined with glucose oxidase (GOx), luminol, and copper sulfate (CuSO_4_), then polymerized by UV irradiation using photoinitiator 2,2‐dimethoxy‐2‐phenylacetophenone (DMPA), followed by glucose incorporation. d) Mechanism of mechano‐chemiluminescence in ^GOx^Gu‐gel: In the unstressed state, GOx is inhibited through multivalent salt‐bridge interactions with Gu^+^ groups in the network. Mechanical stress stretches the polymer chains, reducing the effective multivalency of GOx binding. This partially liberates GOx, enabling it to catalyze glucose oxidation and generate hydrogen peroxide (H_2_O_2_). The H_2_O_2_ then triggers the luminol reaction in the presence of Cu^2+^, resulting in blue light emission.

Mechanoluminescent materials have emerged as powerful stress‐sensing platforms for autonomous light emission in response to mechanical stimuli.^[^ ^1^ ^]^ This unique capability enables real‐time, non‐contact visualization of spatiotemporal stress distributions without requiring external light sources, offering significant advantages for structural health monitoring, wearable sensors, and dynamic stress analysis in engineering and biomedical applications.^[^ ^1^ ^]^ Mechanoluminescent systems developed thus far, consisting of inorganic composites^[^ ^2^ ^]^ or organic solids,^[^ ^3^ ^]^ generate light through various mechanisms: deformation‐induced electronic transitions via lattice or molecular orbital modulation;^[^ ^4^ ^]^ piezoelectric effects leading to direct excitation or interfacial charge separation;^[^ ^5^ ^]^ triboelectric carrier injection and recombination at material interfaces;^[^ ^6^ ^]^ and fracto‐mechanoluminescence during material fracture.^[^ ^7^ ^]^ Polymeric materials, including gel materials, that induce chemiluminescence upon covalent bond scission of specific mechanophore units have also been reported,^[^ ^8^ ^]^ with bis(adamantyl)‐1,2‐dioxetane^[^ ^9^ ^]^ serving as virtually the only mechanophore example employed in these systems. All these mechanoluminescent mechanisms convert a portion of the applied mechanical energy into light emission, functioning instantaneously under dynamic mechanical loads such as impact, deformation, or fracture (**Figure** **1**a). Under static loading conditions, however, where both mechanical and energetic equilibrium are established, the system possesses no available energy to sustain light emission (Figure 1a). This constraint prevents real‐time visualization of stress distributions within materials in load‐bearing applications, where understanding sustained deformation is crucial. To overcome this challenge and achieve mechanoluminescence under static mechanical loads, a fundamentally different mechanism is required.

Here, we report a conceptually new mechano‐chemiluminescent hydrogel (^GOx^Gu‐gel) that achieves sustained light emission under static mechanical loads (Figures 1b,c). Previously, we developed a hydrogel platform (Gu‐gel) with guanidinium (Gu^+^) ions in the gel network polymer that enables reversible on/off switching of enzymes within the gel matrix through the application and removal of mechanical stress.^[^ ^10^ ^]^ In Gu‐gel, the Gu^+^‐appended network polymer binds to the enzymes through multivalent salt‐bridge interactions, suppressing the enzymatic activity (Figure 1d).^[^ ^11^ ^]^ Under mechanical stress, the polymer chains are stretched with restricted conformations^[^ ^12^ ^]^ and reduce the effective multivalency,^[^ ^13^ ^]^ partially liberating the enzymes for their reactions (Figure 1d). Upon stress removal, the polymer chains relax and rebind to the enzymes, inhibiting their activity (Figure 1d). In this system, mechanical energy applied to the gel is stored as stretched polymer chains, which switches the enzymes to their active state. We envisioned that if these activated enzymes could spontaneously induce chemiluminescence, sustained light emission would be possible under static mechanical loads without additional energy input.

As a proof of concept, we designed ^GOx^Gu‐gel (Figure 1c) containing a gel network composed of *N*,*N*‐dimethylacrylamide (DMA) and a Gu^+^‐functionalized acrylamide derivative (GuAA), with glucose oxidase (GOx), glucose, luminol, and copper sulfate (CuSO_4_) incorporated in the gel matrix. Upon mechanical stress, GOx is activated to catalyze glucose oxidation, generating hydrogen peroxide (H_2_O_2_, Figure 1d),^[^ ^14^ ^]^ which drives the luminol chemiluminescence reaction in the presence of a CuSO_4_ catalyst (Figure 1d).^[^ ^15^ ^]^ Since GOx remains active as long as the gel network polymer is stretched, chemiluminescence occurs even under static mechanical loads (Figure 1b), persisting until substrates are eventually consumed. This mechanoluminescence process proceeds without material deterioration, enabling repeated mechanical responses with original luminescence intensity through simple substrate replenishment.

## 2 Results and Discussion

^GOx^Gu‐gel was synthesized through a two‐step process (Figure 1c). First, DMA (2.0 m), GuAA (0.5 mm), and *N*,*N*'‐methylenebis(acrylamide) (BIS, 5.2 mm) were copolymerized in Na_2_CO_3_/NaHCO_3_ buffer (100 mm, pH 8.7) using 2,2‐dimethoxy‐2‐phenylacetophenone (DMAP, 10 µm) as a photoinitiator in the presence of GOx (100 U mL^−1^, 1 mg mL^−1^), luminol (1 mm), and CuSO_4_ (1 mm). The precursor gel, thus obtained, was then soaked in a Na_2_CO_3_/NaHCO_3_ buffer solution (100 mm, pH 8.7) of glucose (30 mm) at 4 °C for 4 h to yield ^GOx^Gu‐gel. The low temperature (4 °C) was employed to minimize undesired enzyme reactions during the glucose incorporation process. As a reference hydrogel, ^GOx^DMA‐gel, which does not contain Gu^+^ moieties in the gel network polymer, was prepared without using GuAA under otherwise identical conditions. The gel samples were stored at 4 °C without further purification until use.

**Figure 2:**
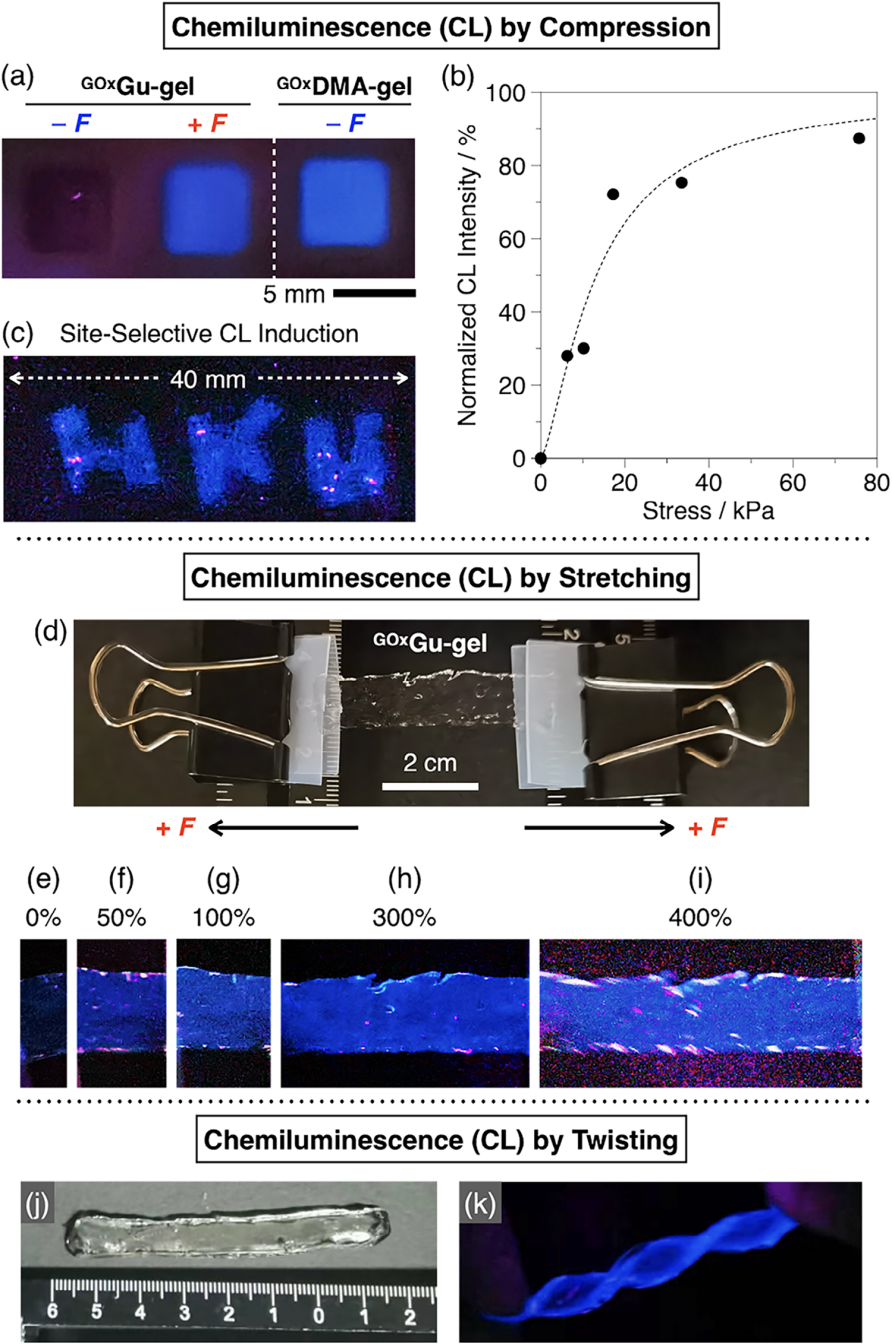
a) Images of hydrogel samples (8 mm × 8 mm × 5 mm) at 25 °C: ^GOx^Gu‐gel left uncompressed for 7 min and after compression at 60% strain for 7 min, and ^GOx^DMA‐gel left uncompressed for 7 min. b) Normalized maximum luminescence intensity of ^GOx^Gu‐gel after compression at different stress values (0–75 kPa), with ^GOx^DMA‐gel luminescence intensity defined as 100%. c) Image of ^GOx^Gu‐gel (40 mm × 20 mm × 8 mm) after compression for 7 min at 25 °C in the shapes of letters “H,” “K,” and “U.” d) Experimental setup for stretching ^GOx^Gu‐gel (original length: 1 cm). e–i) Images of ^GOx^Gu‐gel stretched to 0–400% strain at 25 °C. j,k) Images of ^GOx^Gu‐gel (70 mm × 8 mm × 5 mm) in its original state j) and maintained in a twisted state k).

Compression and tensile tests of ^GOx^Gu‐gel showed fracture stress/strain values of 103 kPa/74% (Figure S10a, Supporting Information) and 13 kPa/423% (Figure S10b, Supporting Information), respectively.^[^ ^16^ ^]^ Based on these mechanical properties, we compressed ^GOx^Gu‐gel with 60% strain at 25 °C. Interestingly, blue luminescence emerged during compression, which was attributed to the luminol reaction (**Figure** **2**a, ^GOx^Gu‐gel + *F*), whereas no luminescence was observed without compression (Figure 2a, ^GOx^Gu‐gel – *F*). In contrast, ^GOx^DMA‐gel started to emit luminescence simply by warming from 4 to 25 °C, without any compression (Figure 2a, ^GOx^DMA‐gel – *F*). This difference between ^GOx^Gu‐gel and ^GOx^DMA‐gel demonstrates that Gu^+^ ions are essential for suppressing GOx enzymatic reactions. Consistently, ^GOx^Gu‐gels prepared with GuAA below 0.1 mm showed luminescence without compression (Figure S11, Supporting Information),^[^ ^16^ ^]^ indicating insufficient enzyme suppression. Meanwhile, gels prepared with GuAA above 1 mm showed limited luminescence even under compression, most likely because denser Gu^+^ moieties in the gel network polymer maintained strong enzyme inhibition (Figure S11, Supporting Information).^[^ ^16^ ^]^ We found that the luminescence intensity of ^GOx^Gu‐gel reached a maximum at 1 mm luminol, while glucose concentration showed minimal influence within the range of 30–750 mm (Figure S12, Supporting Information).^[^ ^16^ ^]^

The luminescence intensity of ^GOx^Gu‐gel increased with applied stress and leveled off at higher stress levels (Figure 2b). This stress‐dependent luminescence response is useful for mapping and monitoring stress distributions in the gel. For example, when ^GOx^Gu‐gel was partially compressed, luminescence was selectively observed in the stressed areas (Figure 2c). Additionally, when a sheet of ^GOx^Gu‐gel was stretched (Figure 2d), luminescence emerged throughout the gel as the strain increased (Figures 2e–i, maintained at strains of 0%, 50%, 100%, 300%, and 400%). Notably, the luminescence persisted under constant strain, demonstrating the capability of ^GOx^Gu‐gel to detect static mechanical loads. When maintained in a twisted form, a strip of ^GOx^Gu‐gel (Figure 2j) exhibited luminescence preferentially at the stress‐concentrated edges (Figure 2k), successfully visualizing the stress distribution within the gel.

**Figure 3:**
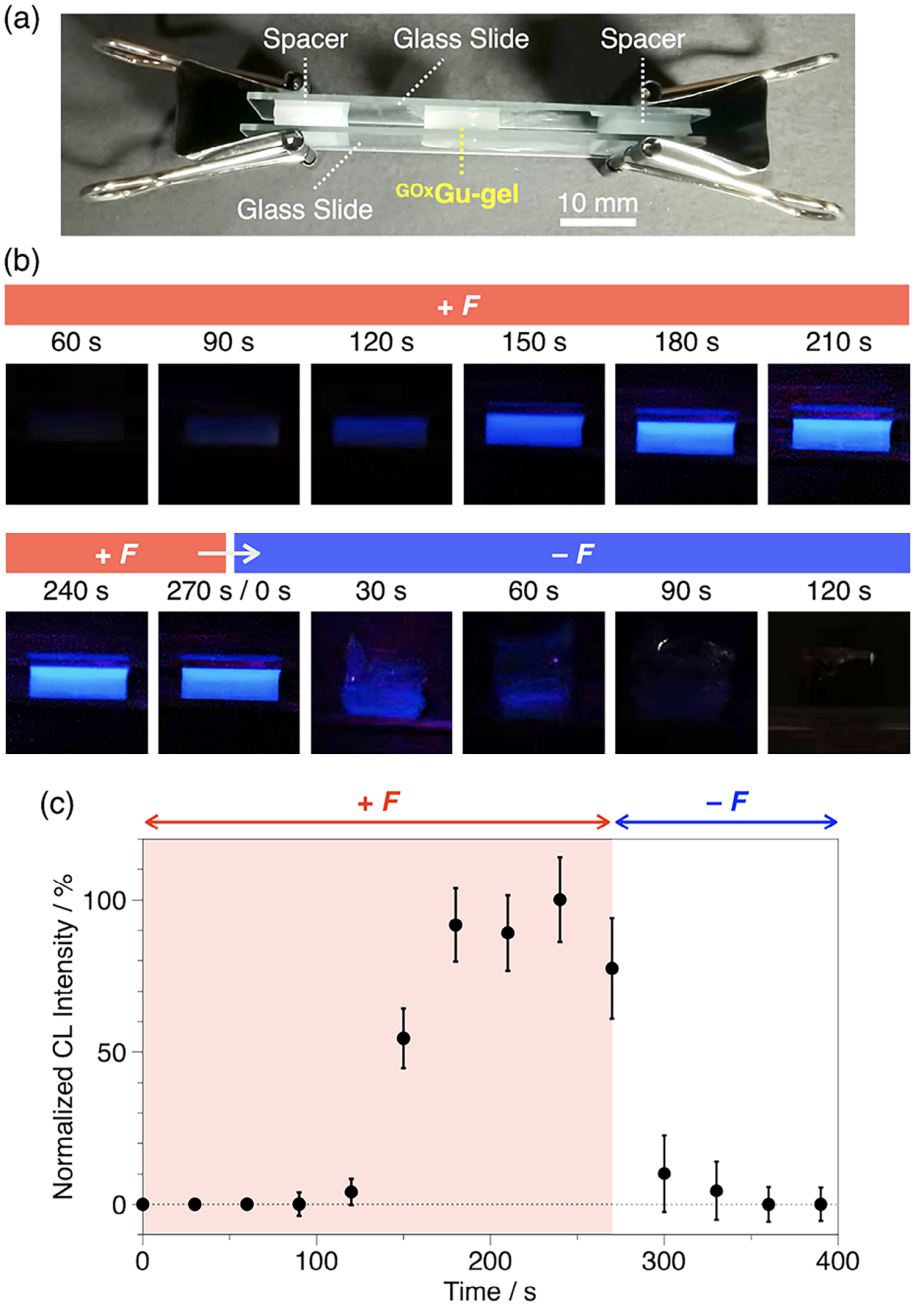
a) Experimental setup for compressing ^GOx^Gu‐gel (5 mm × 5 mm × 6.2 mm) at 60% strain. b) Images of ^GOx^Gu‐gel under sustained compression at 60% strain (60–270 s) and after releasing the compression (0–120 s). c) Normalized luminescence intensity of ^GOx^Gu‐gel under sustained compression at 60% strain (0–270 s) followed by release from compression (270–390 s).

^GOx^Gu‐gel exhibits repeatable luminescence over multiple cycles of mechanical stress. We conducted three compression cycles at 60% strain for 7 min and recorded the maximum luminescence intensity after each period. The gel exhibited luminescence in each cycle; however, the intensity decreased progressively (**Figure** **4**a; 2nd: 62%, 3rd: 42% of initial intensity). Attributing this to luminol consumption, we immersed ^GOx^Gu‐gel in a Na_2_CO_3_/NaHCO_3_ buffer solution of luminol (1 mm) at 4 °C for 4 h after each compression cycle. As a result, the gel showed a luminescent response to three compressions with minimal loss of intensity (Figure 4b; 2nd: 97%, 3rd: 96% of initial intensity). The recovery of luminescence intensity through substrate replenishment indicates that the ^GOx^Gu‐gel system, apart from substrate consumption, maintains its functional integrity throughout the mechanical loading, enzymatic reaction, and luminol oxidation processes. This non‐destructive nature of ^GOx^Gu‐gel complements conventional mechanoluminescent polymer materials that rely on covalent bond scission for light emission, leading to potential applications requiring repeated or prolonged stress monitoring.

We further investigated the time‐course luminescence responses of ^GOx^Gu‐gel to compression. As shown in **Figure** **3**a, a cubic ^GOx^Gu‐gel piece was compressed at 60% strain by sandwiching it between two glass slides with spacers. The luminescence intensity began to increase at 90 s after starting compression and reached nearly maximum intensity at 180 s (Figures 3b,c). This intense emission persisted as long as the compression was maintained, at least until 270 s from the start of compression. The production of gluconic acid as a byproduct of the GOx enzymatic reaction (Figure 1d) did not affect the chemiluminescence during this 90 s period of sustained emission (Figure 3c, 180–270 s), likely because it was neutralized by the Na_2_CO_3_/NaHCO_3_ buffer system. Notably, upon removal of mechanical stress, the luminescence intensity rapidly decreased and almost completely disappeared within 90 s (Figures 3b,c). These observations can be explained by a mechanism in which mechanical stress activates GOx to continuously supply H_2_O_2_, leading to steady‐state luminol chemiluminescence, while stress removal deactivates GOx and results in rapid H_2_O_2_ consumption through the luminol reaction.

**Figure 4:**
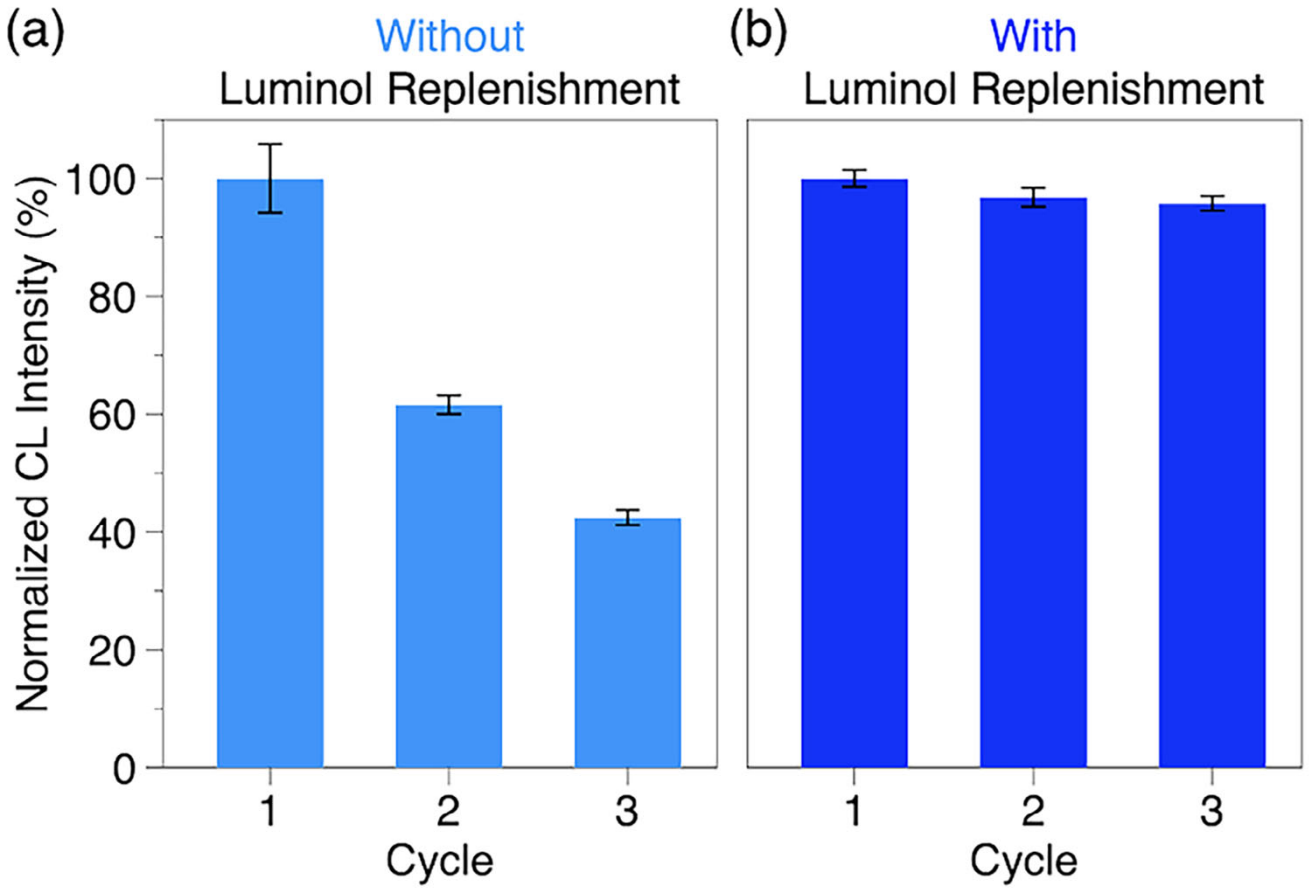
Normalized maximum luminescence intensity of ^GOx^Gu‐gel during each of three successive compression cycles at 60% strain for 7 min: a) without treatment between cycles and b) with the gel immersed in a Na_2_CO3/NaHCO_3_ buffer solution of luminol (1 mm) at 4 °C for 4 h during intervals between cycles.

## 3 Conclusion

In conclusion, we have developed a novel mechano‐chemiluminescent hydrogel system (^GOx^Gu‐gel) that achieves sustained light emission under static mechanical loads. ^GOx^Gu‐gel leverages mechanical stress to activate GOx within the gel matrix, which catalyzes glucose oxidation to generate H_2_O_2_ and subsequently drives the luminol chemiluminescence reaction. This mechanism addresses a critical limitation of conventional mechanoluminescent materials that only function under dynamic loads, enabling visualization of stress distributions in sustained deformation. ^GOx^Gu‐gel maintains its material integrity throughout mechanical cycles and recovers its original luminescent performance through simple substrate replenishment. While the current ^GOx^Gu‐gel demonstrates stress responsiveness within a modest range (10–100 kPa), extending sensitivity to higher stress ranges represents a promising future direction for broader applications, potentially achieved by enhancing mechanical strength through incorporation of a second gel network (double‐network gel)^[^ ^17^ ^]^ with optimized crosslinking density.

## Conflict of Interest

The authors declare no conflict of interest.

## Supporting information



Supporting Information

## Data Availability

The data that support the findings of this study are available from the corresponding author upon reasonable request.
