# Supplementary material for: Mechano‐Chemiluminescent Hydrogel for Sustained Stress Visualization Under Mechanical Equilibrium
Source: Macromol Rapid Commun. 2025 May 21;46(19):2500256. doi: 10.1002/marc.202500256 (PMC12498311; doi:10.1002/marc.202500256)
Supplement: Supplementary file 1 — Supporting Information [file MARC-46-2500256-s001.docx]

Supporting Information

Mechano-Chemiluminescent Hydrogel for Sustained Stress Visualization Under Mechanical Equilibrium

Yiwa Wang, and Kou Okuro*

Table of Contents

**1. General S2**

**2. Synthesis of GuAA S3**

**3. Preparation of ^GOx^Gu-gel and ^GOx^DMA-gel S9**

**4. Mechanical Tests S10**

**5. Mechano-chemiluminescence Measurements S11**

1. General

^1^H and ^13^C nuclear magnetic resonance spectra (NMR) spectra were recorded on a Bruker type AVANCE III 400 spectrometer, where chemical shifts for ^1^H NMR spectroscopy were determined with respect to non-deuterated solvent residues; CHCl_3_ and DMSO, and those for ^13^C NMR spectroscopy were determined with respect to CHCl_3_ and DMSO. Electrospray ionization mass (ESI-MS) spectrometry was performed on a Bruker Daltonics Impact II QTOF spectrometer. Normal-phase column chromatography was performed using DAVISIL silica gel (particle size 40–63 *μ*m). Photoirradiation was carried out with a Forensic Alternate LED light source model OR-GYD70 (*λ* = 370 nm). For pH measurements, Horiba compact pH meter model LAQUAtwin pH-22 was used. Compression and tensile tests were operated on an A&D model MCT-2150 force tester at a constant speed of 10 mm min^–1^ (cylindrical gels: 20 mm diameter, 6 mm height) and 100 mm min^–1^ (dumbbell-shaped gels: test section 2 mm thickness, 15 mm length and 5 mm width), respectively. Luminescence images of gels were captured using a digital camera with ISO-1600 and shutter speed of 10 s.

Unless otherwise noted, reagents and solvents were used as received from commercial sources without further purification. Glucose oxidase and glucose were purchased from Macklin. *N*,*N*-Dimethylacrylamide (DMA), *N*,*N*'-methylenebis(acrylamide) (BIS), and 2,2-dimethoxy-2-phenylacetophenone (DMPA) were purchased from Bide Chemical. Luminol was purchased from TCI. Copper sulfate (CuSO_4_) was purchased from Aladdin.

2. Synthesis of GuAA


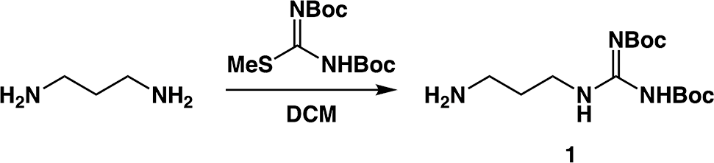


**Compound 1.** To a DCM (32 mL) solution of 1,3-propanediamine (1.86 g, 25.2 mmol) was dropwise added 1,3-di-Boc-2-methylisothiourea (2.04 g, 7.0 mmol), and the mixture was stirred for 90 min at room temperature. Then, the reaction mixture was successively washed with water and brine. An organic extract separated was dried over Na_2_SO_4_ and evaporated to dryness under reduced pressure, affording **1** as a white solid (2.05 g, 92%). ^1^H NMR (400 MHz, CDCl_3_): *δ* 8.35 (t, *J* = 6.9 Hz, 1H; N*H*), 3.46 (t, *J* = 6.8 Hz, 2H; NHC*H*_2_), 2.73 (t, *J* = 6.7 Hz, 2H; C*H*_2_NH_2_), 1.66 (q, *J* = 6.8 Hz, 2H; NHCH_2_C*H*_2_CH_2_NH_2_), 1.44–1.39 (s, 18H; C(C*H*_3_)_3_). ^13^C NMR (101 MHz, CDCl_3_): *δ* 163.55, 156.22, 153.20, 83.00, 79.15, 39.48, 38.43, 32.79, 28.26, 28.04. ESI-MS: m/z 317.22 ([M + H]^+^, calcd: 317.22), 261.16 ([M – C(CH_3_)_3_ + H]^+^, calcd: 261.16).


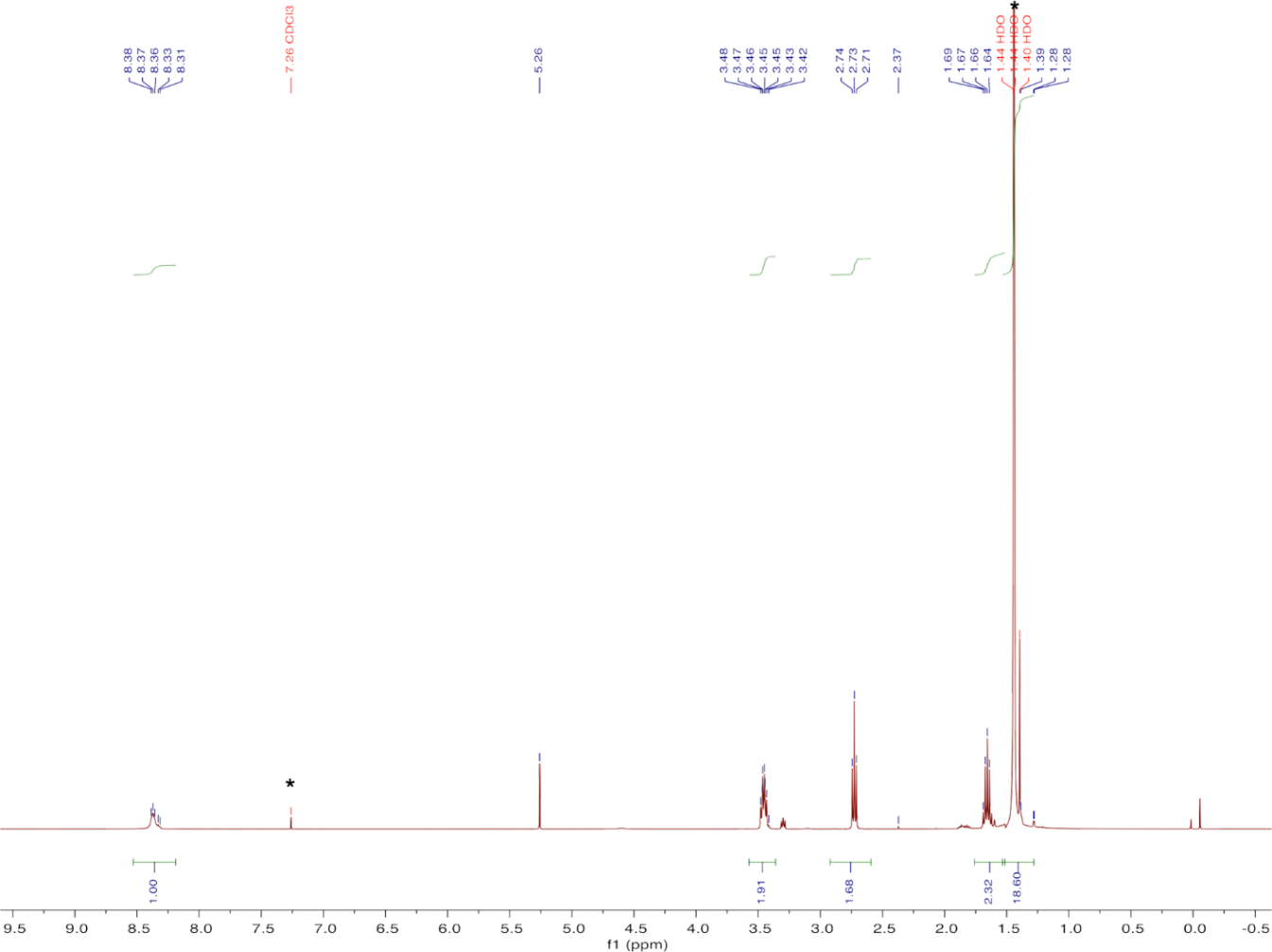


**Figure S1.** ^1^H NMR spectrum of **1** in CDCl_3_ at 25 °C. The signals marked with an asterisk at *δ* 7.26 and 1.44 ppm are due to partially non-deuterated residue of CDCl_3_ and water, respectively.


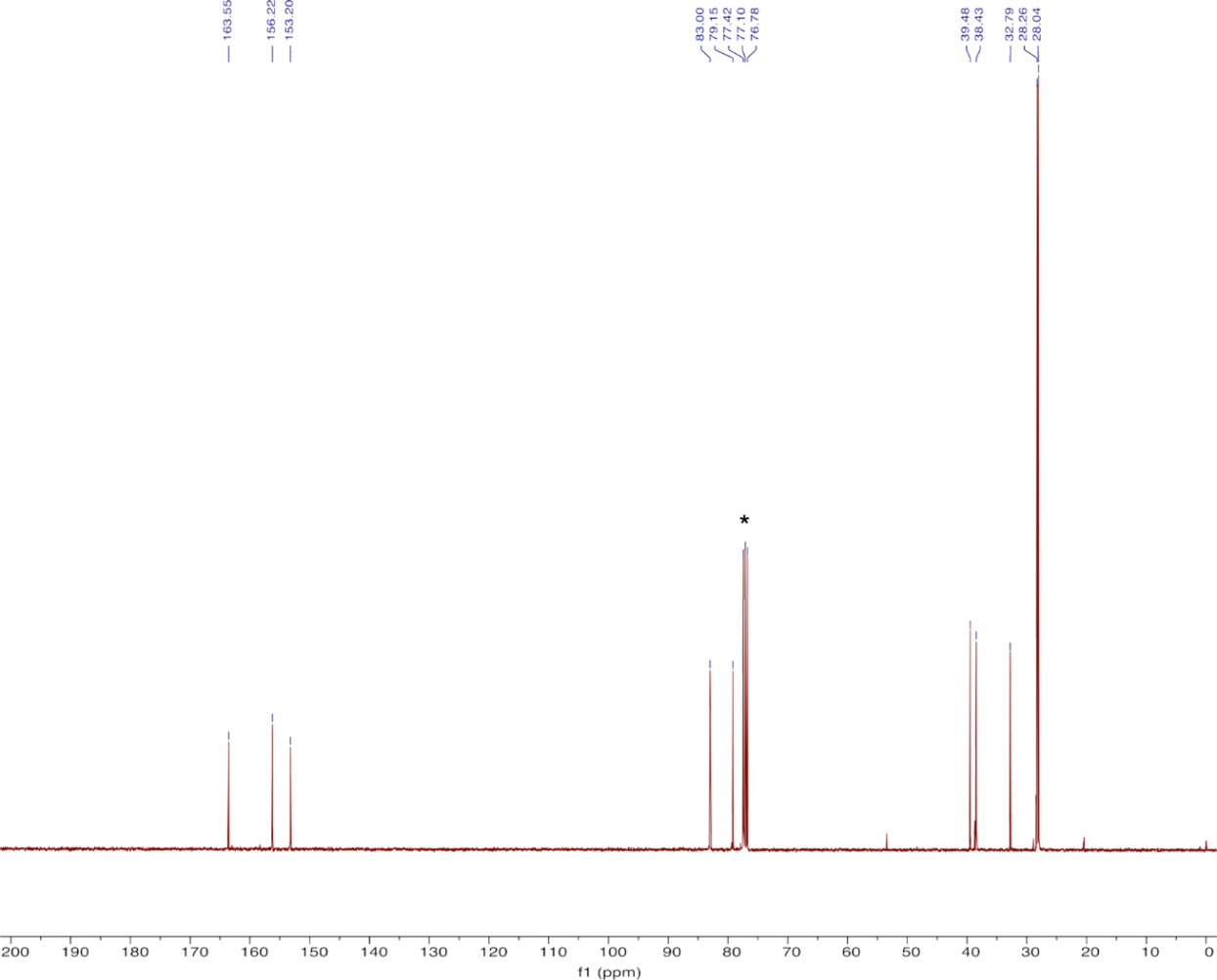


**Figure S2.** ^13^C NMR spectrum of **1** in CDCl_3_ at 25 °C. The signals marked with an asterisk at *δ* 77.10 ppm are due to CDCl_3_.


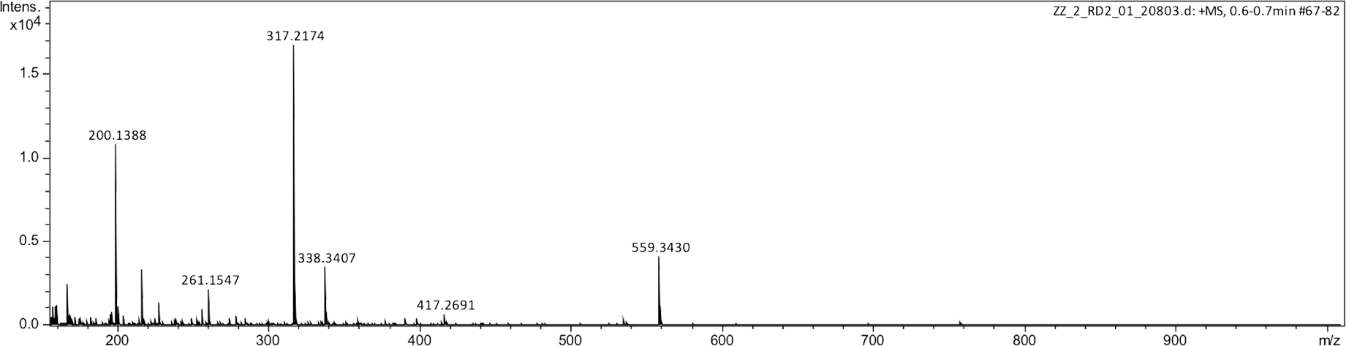


**Figure S3.** ESI-MS spectrum of **1**.


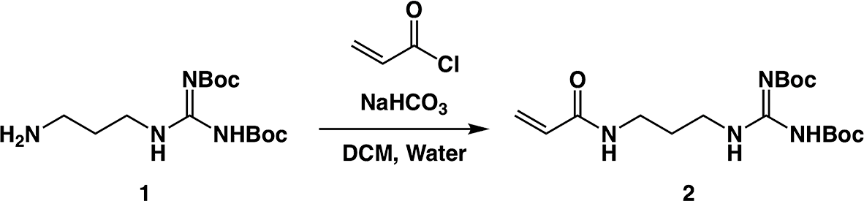


**Compound 2.** To a DCM (10 mL) solution of **1** (2.05 g, 6.48 mmol) and saturated NaHCO_3_ (8 mL) was dropwise added acryloyl chloride (1.17 g, 13.0 mmol) at 0 °C. The reaction mixture was allowed to warm to room temperature and further stirred for 80 min at room temperature. The mixture was diluted with DCM and successively washed with water and brine. An organic extract separated was dried over Na_2_SO_4_ and evaporated to dryness under reduced pressure. The residue was chromatographed on silica gel with hexane/AcOEt (2/8) as an eluent to allow isolation of **2** as a white solid (2.06 g, 87%). ^1^H NMR (400 MHz, CDCl_3_): *δ* 8.41 (t, *J* = 6.6 Hz, 1H; OCN*H*CH_2_CH_2_CH_2_NH), 7.87 (t, *J* = 6.4 Hz, 1H; OCNHCH_2_CH_2_CH_2_N*H*), 6.22 (d, *J* = 6.2 Hz, 2H; C*H*_2_CH), 5.53–5.45 (m, 1H; C*H*CH_2_), 3.42 (d, *J* = 5.9 Hz, 2H; OCNHCH_2_CH_2_C*H*_2_NH), 3.26 (d, *J* = 6.0 Hz, 2H; OCNHC*H*_2_CH_2_CH_2_NH), 1.62 (m, 2H; OCN*H*CH_2_C*H*_2_CH_2_NH), 1.44 (s,18H; C(C*H*_3_)_3_). ^13^C NMR (101 MHz, CDCl_3_) *δ* 165.50, 162.95, 157.18, 153.05, 131.75, 125.31, 83.44, 79.47, 37.07, 34.83, 29.66, 28.24, 27.98. ESI-MS: m/z found: 371.23 ([M + H]^+^, calcd: 371.23), 315.17 ([M – C(CH_3_)_3_ + H]^+^, calcd: 315.17).


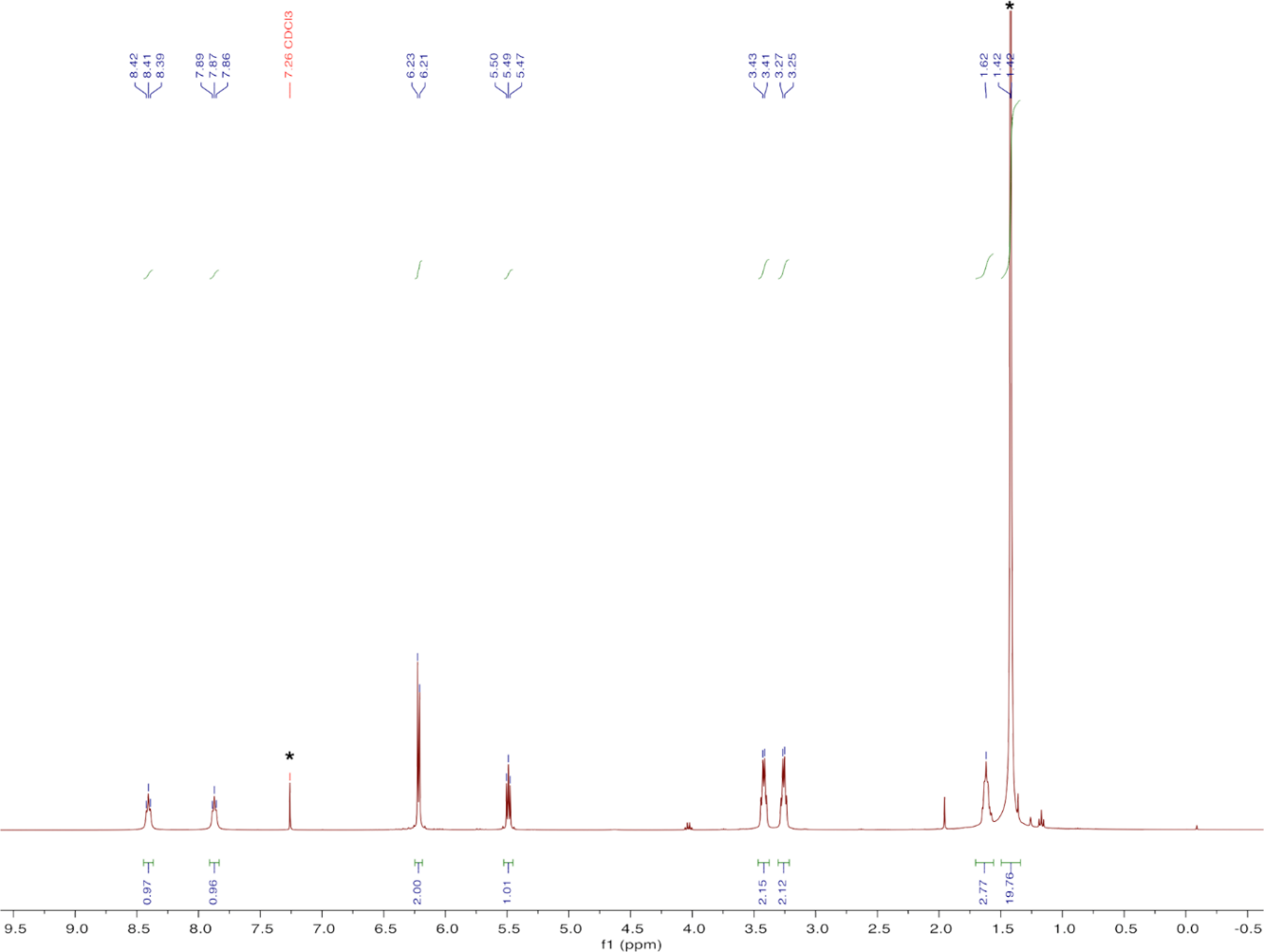


**Figure S4.** ^1^H NMR spectrum of **2** in CDCl_3_ at 25 °C. The signals marked with an asterisk at *δ* 7.26 and 1.42 ppm are due to partially non-deuterated residue of CDCl_3_ and water, respectively.

**
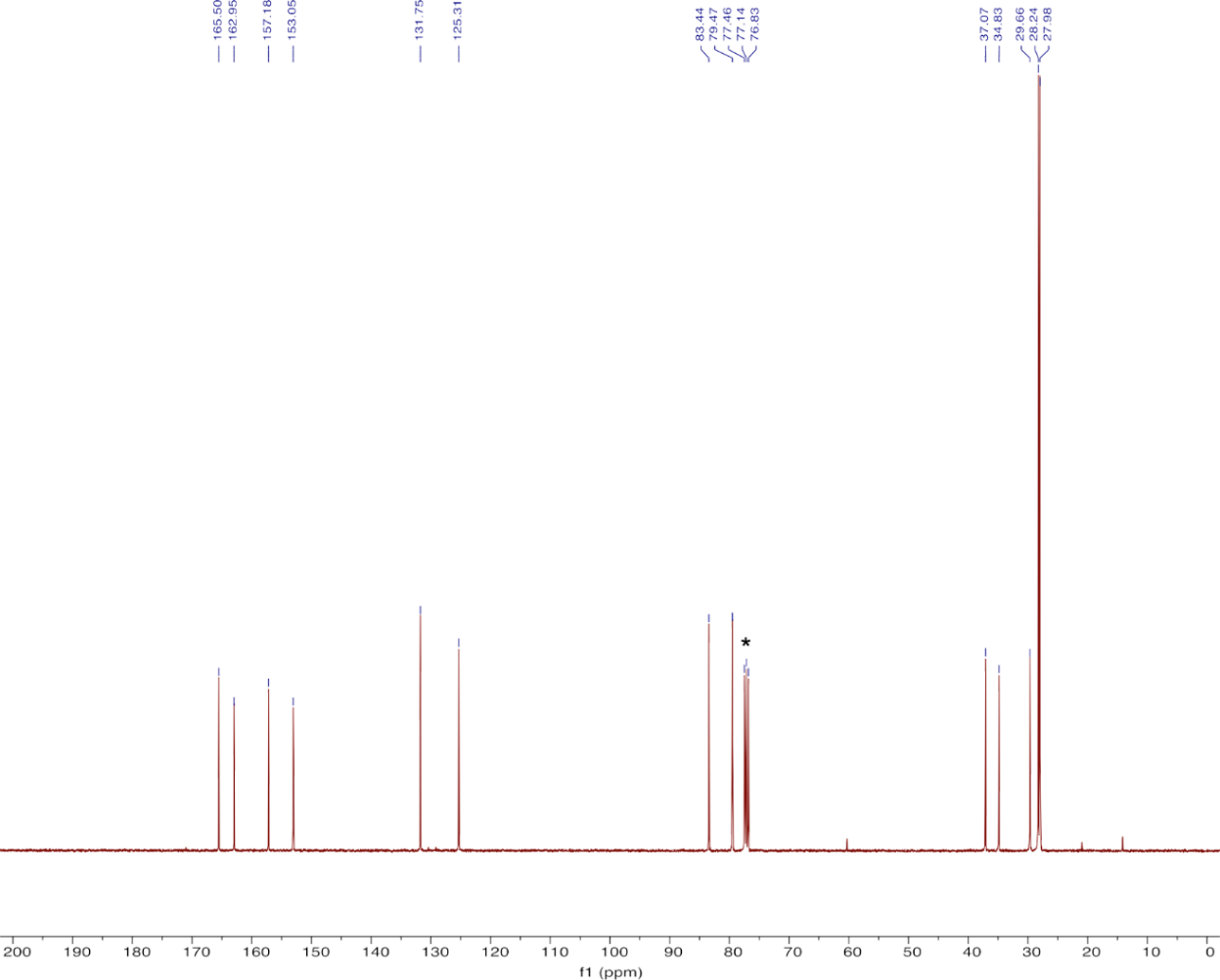
**

**Figure S5.** ^13^C NMR spectrum of **2** in CDCl_3_ at 25 °C. The signals marked with an asterisk at *δ* 77.14 ppm are due to CDCl_3_.

**
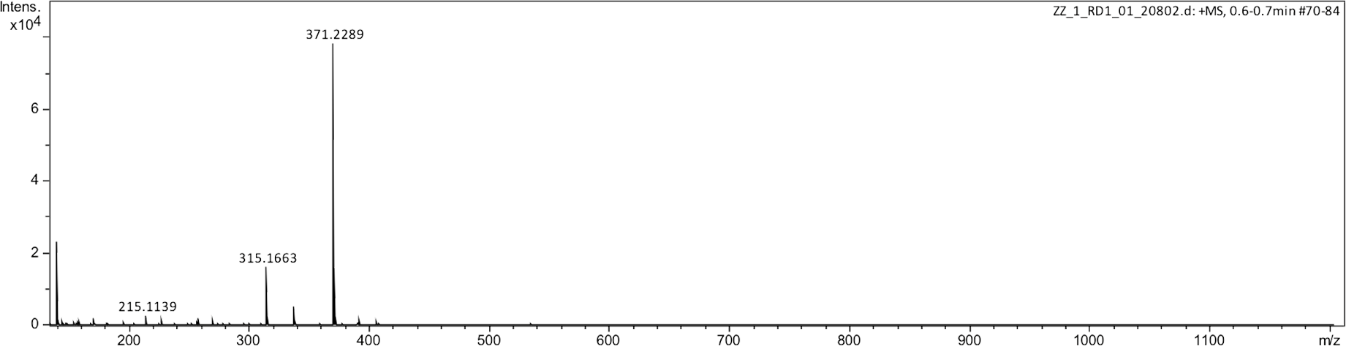
**

**Figure S6.** ESI-MS spectrum of **2**.

**
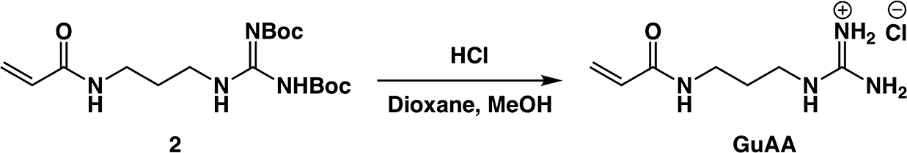
**

**GuAA.** Compound **2** (2.0 g, 5.4 mmol) was dissolved in a mixture of 1,4-dioxane solution of HCl (4 M, 10 mL) and MeOH (10 mL), and the mixture was stirred for 12 h at room temperature. Then, the reaction mixture was reprecipitated with diethyl ether and evaporated to dryness under reduced pressure to allow the isolation of **GuAA** as a white solid (890 mg, 81%). ^1^H NMR (400 MHz, DMSO): *δ* 7.68 (s, 1H; OCN*H*CH_2_CH_2_CH_2_NH), 7.12 (s, 1H; OCNHCH_2_CH_2_CH_2_N*H*), 5.74–5.31 (m, 2H; C*H*_2_CH), 4.94–4.91 (dd, *J* = 6.0 Hz, 1H; CH_2_C*H*), 2.51–2.45 (m, 2H, OCNHCH_2_CH_2_C*H*_2_NH), 1.86–1.84 (t, 2H; OCNHC*H*_2_CH_2_CH_2_NH), 0.99–0.96 (t, 2H; OCN*H*CH_2_C*H*_2_CH_2_NH). ^13^C NMR (101 MHz, DMSO): *δ* 165.31, 157.38, 132.15, 125.55, 48.50, 36.27, 29.00. ESI-MS: m/z found: 171.12 ([M – Cl]^+^, calcd: 171.12).


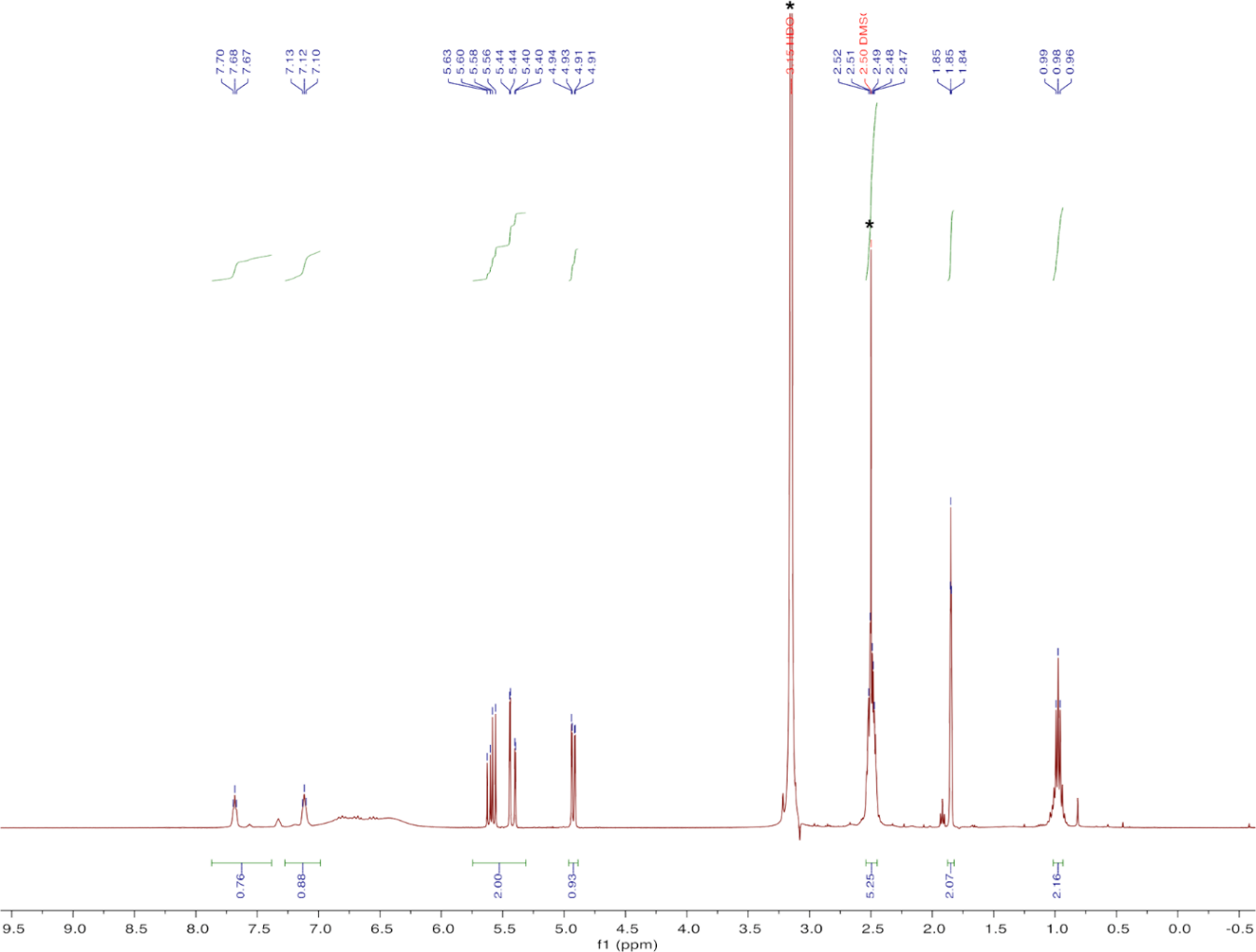


**Figure S7.** ^1^H NMR spectrum of **GuAA** in *d*_6_-DMSO at 25 °C. The signals marked with an asterisk at *δ* 2.50 and 3.15 ppm are due to partially non-deuterated residue of DMSO and water, respectively.


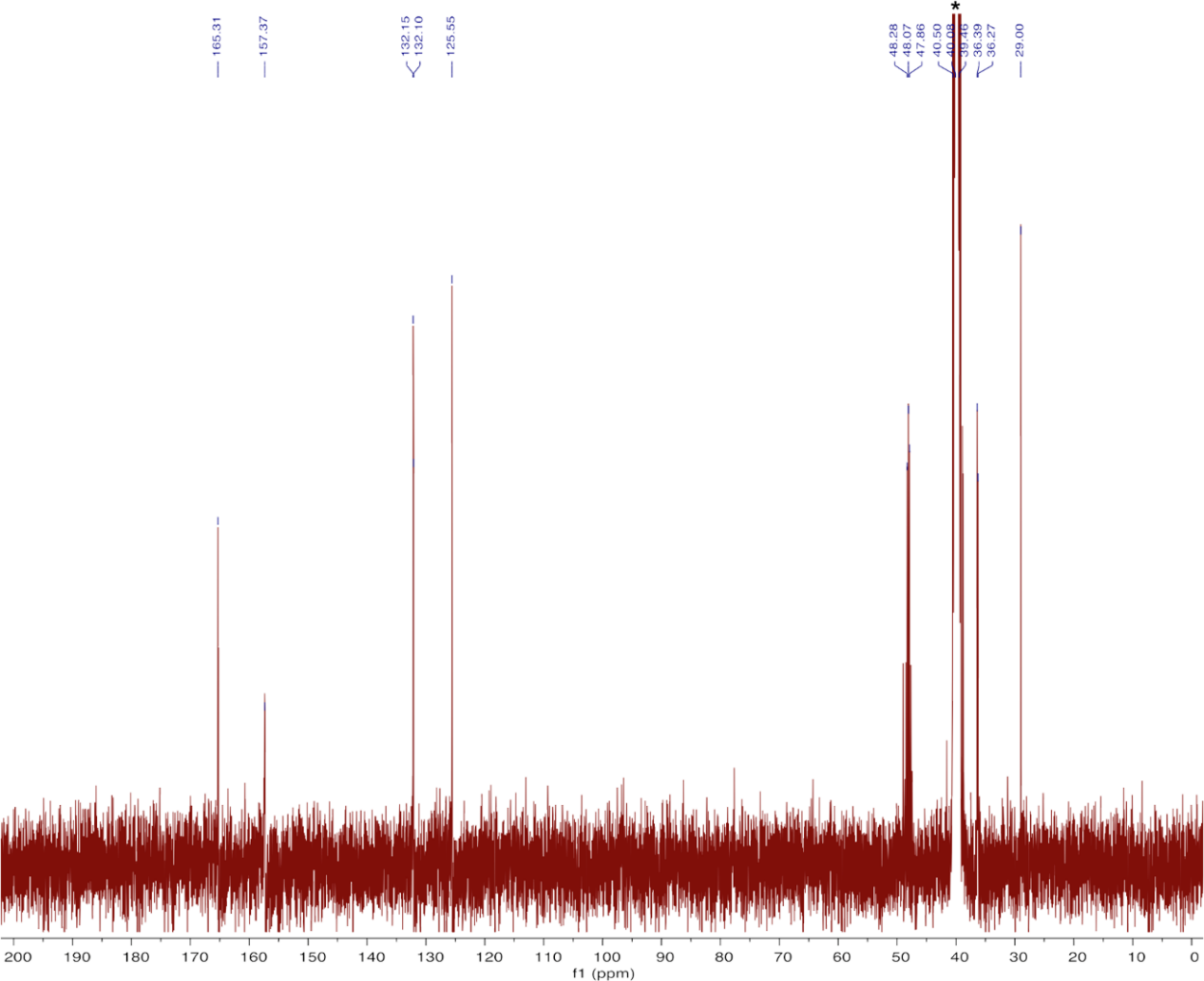


**Figure S8.** ^13^C NMR spectrum of **GuAA** in *d*_6_-DMSO at 25 °C. The signal marked with an asterisk at *δ* 39.5 is due to DMSO.

**
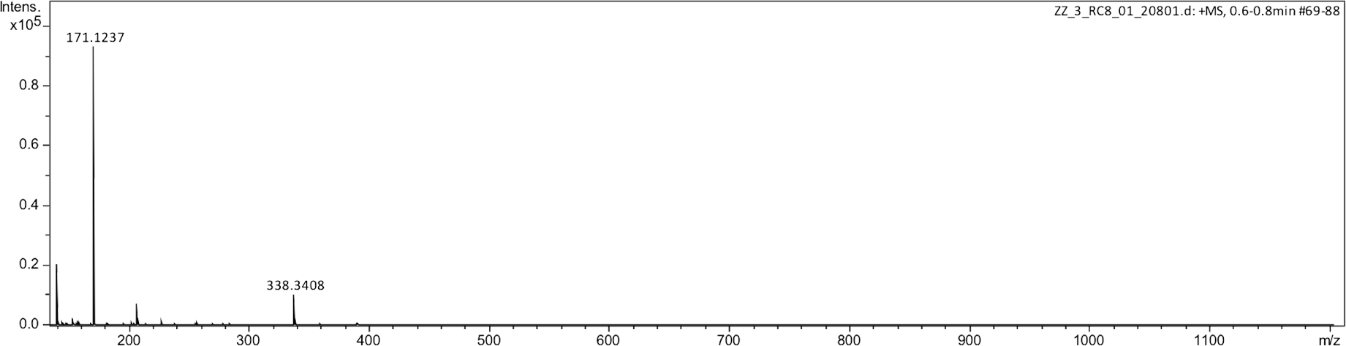
**

**Figure S9.** ESI-MS spectrum of **GuAA**.

3. Preparation of ^GOx^Gu-gel and ^GOx^DMA-gel

To a Na_2_CO_3_/NaHCO_3_ buffer solution (100 mM, pH 8.7) of glucose oxidase (100 U/mL, 1 mg/mL) and GuAA (0.5 mM), CuSO_4_ (1 mM), luminol (1 mM), *N*,*N*-dimethylacrylamide (DMA, 2.0 M), *N*,*N*'-methylenebis(acrylamide) (BIS, 5.2 mM), and 2,2-dimethoxy-2-phenylacetophenone (DMAP, 10 µM) were successively added. The resulting mixture was exposed to UV light at 370 nm for 30 min. The resulting gel was then soaked in a Na_2_CO_3_/NaHCO_3_ buffer solution (100 mM, pH 8.7) of glucose (30 mM) at 4 °C for 4 h to yield ^GOx^Gu-gel. Reference ^GOx^DMA-gel was prepared without GuAA under otherwise identical condition. The gel samples were stored at 4 °C without further purification until use.

4. Mechanical Tests


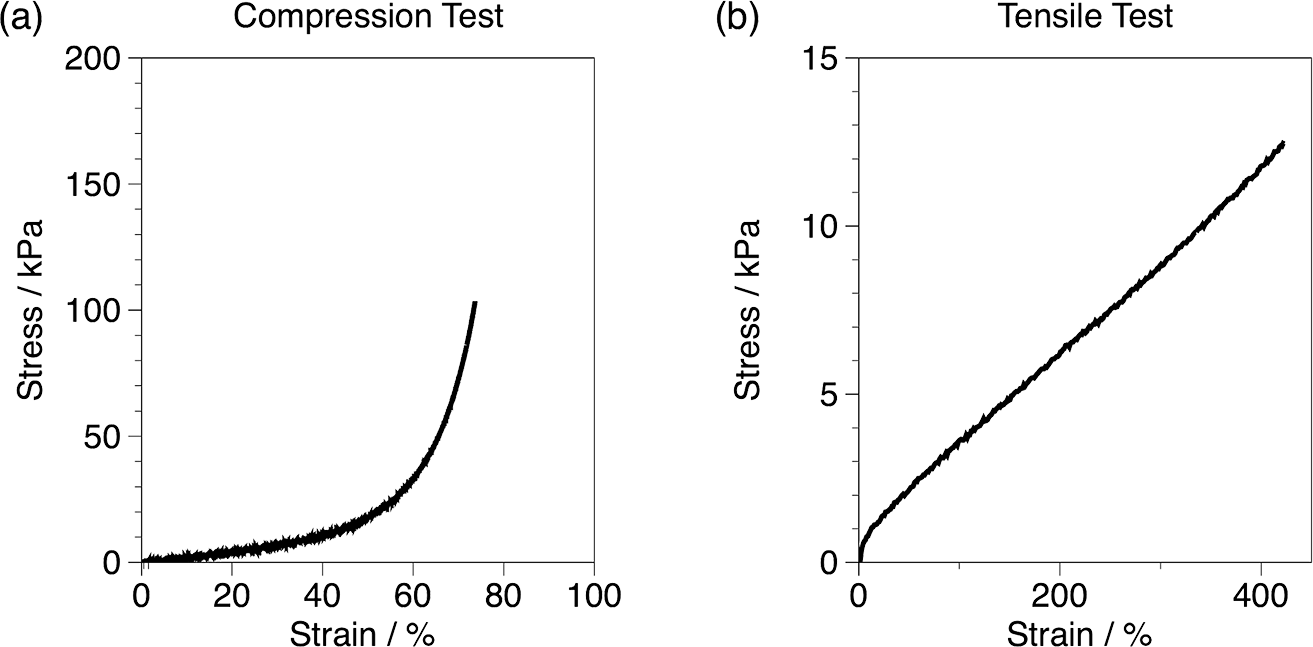


**Figure S10.** Stress-strain curves of ^GOx^Gu-gel in (a) a compression test and (b) a tensile test.

5. Mechano-chemiluminescence Measurements

**
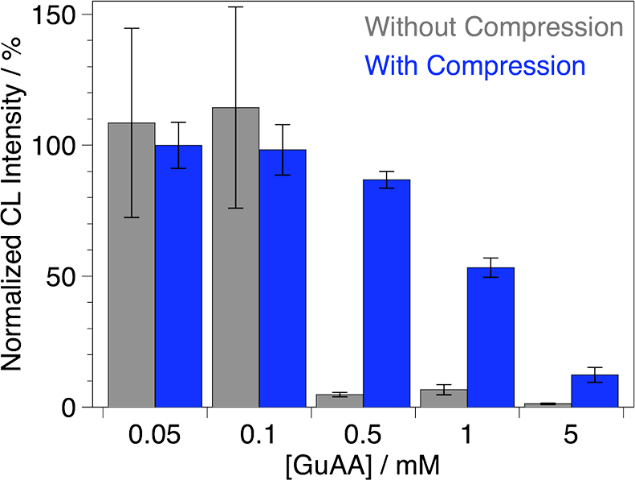
**

**Figure S11.** Normalized maximum luminescence intensity of ^GOx^Gu-gel prepared with different concentrations of GuAA (0.05–5 mM) before and after compression at 60% strain, with luminescence intensity of uncompressed ^GOx^DMA-gel defined as 100%.

**
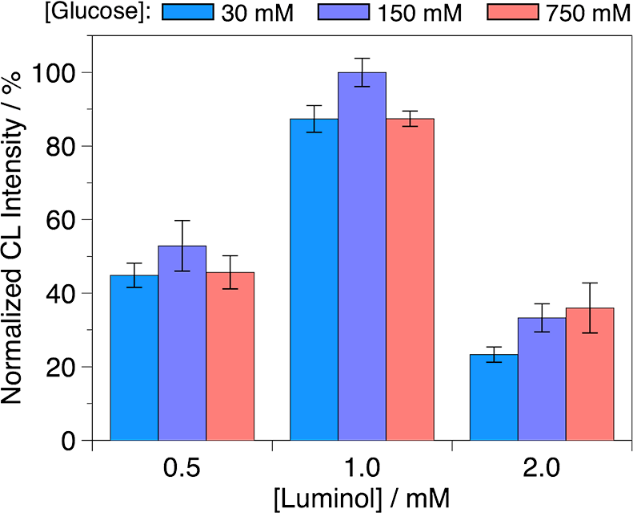
**

**Figure S12.** Normalized maximum luminescence intensity of ^GOx^Gu-gel after compression at 60% strain, prepared with different concentrations of glucose (30, 150, and 750 mM) and luminol (0.5, 1, and 2 mM). The maximum intensity observed in this dataset was defined as 100%.
